# Numerical evaluation and optimization of high sensitivity Cu_2_CdSnSe_4_ photodetector

**DOI:** 10.1016/j.heliyon.2024.e36821

**Published:** 2024-08-23

**Authors:** Md. Choyon Islam, Bipanko Kumar Mondal, Md. Alamin Hossain Pappu, Jaker Hossain

**Affiliations:** a Solar Energy Laboratory, Department of Electrical and Electronic Engineering, University of Rajshahi, Rajshahi, 6205, Bangladesh; b Department of Electrical and Electronic Engineering, Begum Rokeya University, Rangpur, Rangpur, 5400, Bangladesh

**Keywords:** CdS, Cu_2_CdSnSe_4_ (CCTSe), MoS_2_, Responsivity, Detectivity

## Abstract

Copper cadmium tin selenide (Cu_2_CdSnSe_4_) based photodetector (PD) has been explored with the solar cell capacitance simulator (SCAPS-1D). Herein, cadmium sulfide (CdS) and molybdenum disulfide (MoS_2_) are used as a window and back surface field (BSF) layers, respectively. The physical attributes, such as width, carrier density and bulk defects have been adjusted to attain the optimal conditions. In an optimized environment, the performance parameters of the Cu_2_CdSnSe_4_ (CCTSe) PD e.g. open circuit voltage (V_OC_), short circuit current (J_SC_), responsivity, and detectivity are determined as 0.76 V, 45.57 mA/cm^2^, 0.72 A/W and 5.05 × 10^14^ Jones, respectively without a BSF layer. After insertion of the BSF layer, the performance of the CCTSe PD is significantly upgraded because of the production of high built-in potential which rises the magnitude of V_OC_ from 0.76 V to 0.84 V. For this reason, the responsivity and detectivity of CCTSe PD are also grows with the value of 0.84 A/W and 2.32 × 10^15^ Jones, respectively that indicate its future potential.

## Highlights

• Highly efficient Cu_2_CdSnSe_4_ photodetector (PD) has been designed and analyzed.
• Herein, CdS and MoS_2_ work as window and BSF, respectively.
• The PD exhibits a voltage of 0.84 V and current of 45.57 mA/cm^2^, respectively.
• Cu_2_CdSnSe_4_ PD shows a responsivity of 0.84 A/W and detectivity of 2.32 × 10^15^ Jones.
• Cu_2_CdSnSe_4_ PD is highly potential in the field of photodetector.

## 1 Introduction

A photodetector is a specialized optoelectronic gadget which detects impinged light or other forms of electromagnetic radiation, such as Ultraviolet (UV), visible, and infrared (IR) radiation into electrical signal. Light exhibits a range of characteristics determined by its wavelength. Ultraviolet light covering 0.25 μm–0.4 μm, visible light spanning 0.45 μm–0.8 μm, encompasses the colors of the spectrum visible to the human eye. Infrared (IR) light with wavelengths from 0.9 μm to 1.7 μm and beyond, includes near-infrared, mid-infrared, and far-infrared regions [1]. In response to the multispectral characteristics of light signals, photodetectors have evolved to efficiently identify UV, visible, and infrared light, exhibiting qualities such as fast response, wide bandwidth, ample area, cost-effectiveness, and energy efficiency [[2], [3], [4], [5]]. Photodetectors find widespread applications in both commercial and scientific domains, playing essential roles in fiber optic data transmission, flame and fire detection, ambient surveillance, day-night vision, optical communication, remote control, secure space-to-space communications, biochemical detection, medical diagnostics, aviation, missile warning systems, target identification, missile warning, and various other fields [[6], [7], [8], [9]]. Nowadays, silicon-based PDs are commercially ubiquitous worldwide. But Si-based PDs have some boundaries such as the need for ultra-high vacuum environments, and high voltage supplies, along with low responsivity and detectivity [10,11]. To address these issues and pursue advancing and efficient PD performance over the years, researchers are focusing on improving the responsivity and detectivity of PD. Chalcopyrite semiconductors that belong to the I_2_-II-IV-VI_4_ group contain some exceptional properties that are useful for a perfect PD. Some research has been conducted on PDs based on materials like CZTSSe (copper zinc tin sulphur selenide), revealing promising responsivity of 18 mA/W and detectivity of 10^9^ Jones [12]. Moreover, CZTS (copper zinc tin selenide) PD showcases a responsivity of 0.41 A/W and a detectivity of 10^12^ Jones [13]. Furthermore, CZTSSe/CdS-based hetero-junctions indicate a responsivity of 0.39 A/W and detectivity of 2.04 × 10^11^ Jones within the wavelength range of 300 nm–1100 nm [14]. In this article, a novel Cu_2_CdSnSe_4_-based PD has been introduced subjecting to numerical investigation using the widely employed software SCAPS-1D. Cu_2_CdSnSe_4_, a quaternary chalcogenide forms one of the chalcopyrite-like structures, namely stannite, and the compound has a direct bandgap [15,16]. The selection of Cu_2_CdSnSe_4_ as the absorber layer contributes to specific properties. The properties are a high Seebeck coefficient or thermopower, a higher absorption coefficient (10^5^ cm^−1^) in the visible light spectrum [17,18], a low thermal conductivity, and a very high thermoelectric figure of merit (ZT) value [15]. Furthermore, the electrical conductivity of CCTSe can be modified by using a suitable dopant such as Cu. The suitable dopant enhances the electrical conductivity and diminish thermal conductivity [15]. Moreover, owing to the presence of complex structure and multiple components of CCTSe compound also shows low thermal conductivity [19]. The improved power factor makes it promising and suitable in the field of phonon-glass and electron crystal [20]. These properties of CCTSe make it appropriate for applications in different areas such as thermoelectric, PV cells and photosensors. To the best of our knowledge, Cu_2_CdSnSe_4_ material-based photodetector has not been fabricated before despite its excellent properties. The unique properties of this material lie in its ability to enhance responsivity and detectivity. These unique properties of this material and the ability to work at higher temperature make this compound efficient in the photodetector field.

In addition, CdS is a widely studied window layer or n-type semiconductor with an absorber layer due to its tunable wide optical bandgap, elevated carrier density, and excellent transmission properties. Noticeably, it exhibits remarkable stability under continuous exposure to light, contributing to its popularity in various scientific investigations globally [[21], [22], [23]]. To extend into light absorption in photodetectors, it's crucial to minimize unwanted light reflection at the air-top layer interface. This is achieved through methods like applying anti-reflection coatings or using materials with lower refractive indices, effectively enhancing the overall efficiency of the photodetector [24].

MoS_2_, a dichalcogenide semiconductor compound, has been used as a BSF layer to form a barrier potential and reduce surface recombination with absorber layer. It contains some exceptional features like tunable direct bandgap, with a high absorption coefficient of 10^6^ cm^−1^ and an extended diffusion length of 1 μm is used as a BSF layer that creates another p-n junction with the absorber and reduces the non-uniformity of photon absorptions [[25], [26], [27], [28]].

Therefore, in this article, a highly efficient CCTSe-based novel PD has been proposed where CdS and MoS_2_ are used as a window and BSF layers. The sketched n-CdS/p-Cu_2_CdSnSe_4_/p^+^-MoS_2_ PD is numerically investigated by SCAPS-1D. From this structure, the current-voltage and the PD parameters have been reckoned and also the effect of resistance and temperature on this novel structure has been probed. Herein, the performance of the similar structure as solar cell is also demonstrated.

## 2 Methodology and simulation parameters

**Fig. 1:**
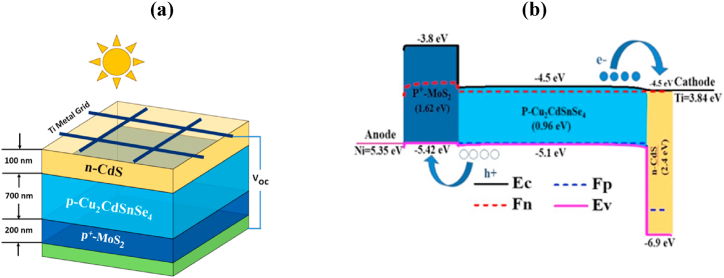
(a) Illustrative diagram (b) Electronic band lay out of n-CdS/p-Cu_2_CdSnSe_4_/p^+^-MoS_2_ PD.

Fig. 1 explores the schematic presentation and band layout of the n-CdS/p-Cu_2_CdSnSe_4_/p^+^-MoS_2_ photodetector. Detecting the optical signal by the CCTSe photodetector is performed where sunlight gets in via the n-type CdS layer, and absorption and detection take place in the p-type CCTSe layer as in Fig. 1(a). This p-type CCTSe layer is characterized by an electron affinity (E.A.) and an ionization potential (I.P.) of 4.5 eV and of 5.1 eV, respectively. The consistency between p-type CCTSe and n-type CdS is established based on their E.A. values of 4.5 eV and I.P. values of 6.9 eV [16]. MoS_2_ layer is initiated to form a beneficial band alignment with p-CCTSe based on sound energy levels outlined in Fig. 1(b). The heterojunction between MoS_2_ and CCTSe is considered advantageous, given that MoS_2_ has an E.A. of 3.8 eV [29]. For effective carrier transfer between electrodes and absorber layers, Ti and Ni metals are strategically chosen as front and rear contact layers.

The SCAPS-1D device, constructed by M. Burgelman et al., is exerted to conceptually study the n-CdS/p-Cu_2_CdSnSe_4_/p^+^-MoS_2_ PD device [30]. Operating conditions for the simulation include a global air mass of 1.5G spectrum, a single sun illumination, and 300 K as ambient temperature with an incident power of 100 mW/cm^2^. In n-CdS/p-Cu_2_CdSnSe_4_/p^+^-MoS_2_ PD, the impacts of physical parameters like thickness, doping concentration, and defect density are tested using a SCAPS-1D simulator. This simulation software is used to determine the characteristics curve such as J-V and quantum efficiency (QE) of solar cells and PDs. SCAPS-1D simulation software solves the Poisson equations related to electrons and holes in semiconductors [[31], [32], [33]]. These equations are shown below:

(1) $$\frac{\partial^{2}\Psi }{\partial x^{2}}+\frac{q}{\varepsilon }\;\left[p\left(x\right)-n\left(x\right)+N_{D}-N_{A}+\rho_{p}-\rho_{n}\right]=0$$

(2) $$\frac{\partial J_{p}}{\partial x}=q\left\{G_{op}-R\left(x\right)\right\}$$

(3) $$\frac{\partial J_{n}}{\partial x}=q\left\{{-G}_{op}+R\left(x\right)\right\}$$

In this context, *ε*, q, N_A_, and N_D_ are the dielectric constant, charge of electron, ionized acceptors density, and of ionized donors' density, respectively. J_p_ and J_n_ represent holes and electrons current density. Ψ stands for electrostatic potential, while G_op_ and R indicate the total carrier production and recombination rate. Furthermore, p and n indicate the concentrations of free holes and electrons, and ρ_p_ and ρ_n_ signify the holes and electrons distribution, accordingly. Responsivity, R (A/W), and Detectivity, D* (Jones) are two important factors for evaluating a device's performance and sensitivity. The equations of responsivity and detectivity are shown below [34]:

(4) $$Responsivity\left(R\right)=\frac{q\times \eta \times \lambda }{h\times c}$$

(5) $$D\text{etectivity}\left(D^{*}\right)=\frac{R}{\sqrt{2\times q\times J_{0}}}$$

Where q is the charge of the electron, η is QE, λ is incoming photon wavelength, h is Planck's constant, J_0_ is density of dark current and c is the light velocity.

The various parameters of each PD layer used for the computation are revealed in Table 1. The parameters of CdS [17], CCTSe [17,35] and MoS_2_ [29] are collected from the literatures. The optical data of constituent layers are generated from the SCAPS sqrt()_Eg model.

**Table 1:** Input parameters used for simulating n-CdS/p-Cu_2_CdSnSe_4_/p^+^-MoS_2_ photodetector.

Parameters	*n*-CdS [17]	*p*-Cu_2_CdSnSe_4_ [17,35]	*p* + -MoS_2_ [29]
Layer	Window	Absorber	BSF
Thickness (nm)	100	700	200
Bandgap, E_g_ [eV]	2.40	0.96	1.62
Electrons affinity, χ [eV]	4.50	4.5	3.8
Dielectric permittivity (relative)	10.0	9.0	10.07
Effective DOS at CB [cm^−3^]	2.2 × 10^18^	1.7 × 10^16^	2.8 × 10^19^
Effective DOS at VB [cm^−3^]	1.8 × 10^19^	2.2 × 10^16^	1.0 × 10^19^
Electron thermal velocity (cms^−1^)	1.0 × 10^7^	1.0 × 10^7^	1.0 × 10^7^
Hole thermal velocity (cms^−1^)	1.0 × 10^7^	1.0 × 10^7^	1.0 × 10^7^
Electron Mobility μ_n_ [cm^2^V^−1^s ^−1^]	1.0 × 10^2^	20	12
Hole mobility μ_p_ [cm^2^V^−1^s^−1^]	2.5 × 10^1^	20	2.8
Donor density N_D_ [cm^−3^]	1.0 × 10^18^	0	0
Acceptor density N_A_ [cm^−3^]	0	1.0 × 10^16^	1.0 × 10^19^
Total defect density, N_t_ [cm^−3^]	1.0 × 10^15^	1.0 × 10^15^	1.0 × 10^15^

## 3 Result and discussion

### 3.1 Impact of BSF on Cu_2_CdSnSe_4_ photodetector

**Fig. 2:**
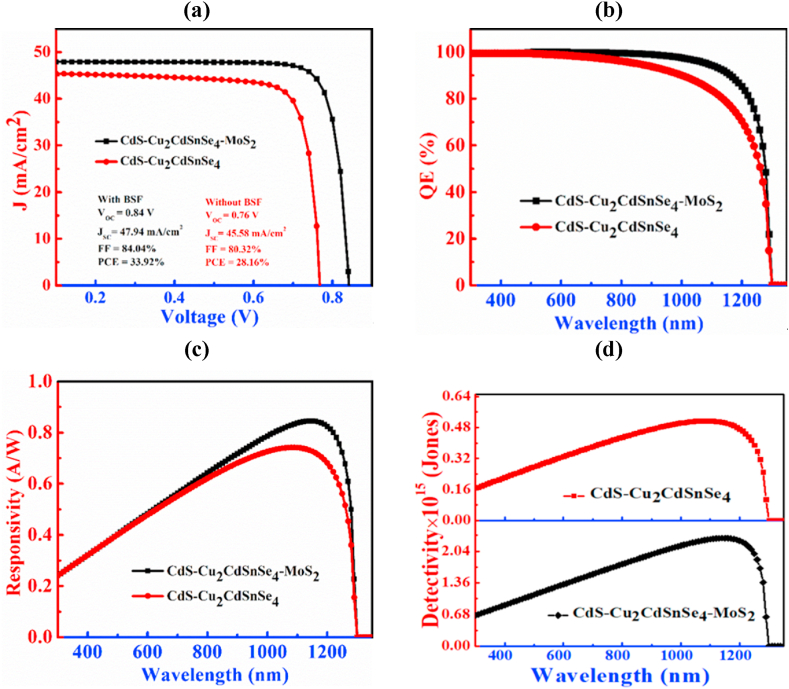
Influence of MoS_2_ as a BSF layer on Cu_2_CdSnSe_4_ Photodetector: (a) J-V, (b) QE, (c) responsivity and (d) detectivity.

Fig. 2 exhibits the importance of the MoS_2_ layer on the CCTSe PD. In Fig. 2(a), the J-V characteristic curves of a CCTSe-based PD with and without a BSF layer is revealed. Here, without a BSF layer, the values of V_OC_ and J_SC_ are recorded as 0.76 V and 45.57 mA/cm^2^, respectively. Adjoining a MoS_2_ layer to the single heterojunction elevates both V_OC_ and J_SC_ to 0.84 V and 47.92 mA/cm^2^, respectively. This is because the MoS_2_ layer creates a p/p^+^ junction with the CCTSe layer that lessens the surface recombination velocity enhances the J_SC_, and raises the built-in potential which is responsible for the increment of V_OC_ [36,37].

The expression of V_OC_ is related to J_SC_ and dark current, J_0_ by

(6) $$V_{oc}\approx \frac{k_{B}T}{q}\ln\frac{J_{sc}}{J_{0}}$$

Where, K_B_ denotes Boltzmann's constant, q represents the electronic charge, and T is the absolute temperature. For a structure incorporating a BSF layer, the V_OC_ is observed to be 0.84 V, with a corresponding J_SC_ of 47.92 mA/cm^2^. The dark current density (J_0_) derived from equation (6), is calculated to be 4.46 × 10^−13^ mA/cm^2^. Conversely, for a structure lacking a BSF layer, the V_OC_ measures 0.76 V, and the J_SC_ is 45.57 mA/cm^2^. In this case, the dark current density (J_0_) is determined to be 9.20 × 10^−12^ mA/cm^2^. Fig. 2(a) also indicates that in the inexistence of the BSF layer, the fill factor (FF) and power conversion efficiency (PCE) are 80.32 % and 28.16 %, respectively. Conversely, the addition of the BSF layer results in improved values of FF of 84.04 % and PCE of 33.92 %.

Fig. 2(b) displays the QE characteristic curves for the CCTSe PD and showcases its performance with and without the BSF layer. Without a BSF layer, the PD exhibits over 99 % photon absorption efficiency in the 300–560 nm range, declining to approximately 50 % at 1260 nm and reaching zero at 1300 nm. Adding a thin BSF layer i.e. MoS_2_ enhances photon absorption, showing over 99 % efficiency from 300 to 500 nm, 100 % from 510 to 590 nm, and maintaining around 99 % from 600 to 890 nm. MoS_2_ demonstrates compatible band alignment with the CCTSe absorber layer in photodetectors. This alignment selectively impedes the flow of majority charge carriers, directing them back towards the front contact electrode. Consequently, recombination is minimized, leading to enhanced performance of CCTSe photodetectors [38,39].

In Fig. 2(c), the graph depicts variation in responsivity with wavelength in presence or absent of MoS_2_ BSF layer. Within a single heterojunction, the CCTSe PD shows a responsivity of 0.72 A/W at a wavelength of 1150 nm. After adding MoS_2_ as a BSF layer, the PD responsivity improves reaching to 0.84 A/W at a wavelength of 1150 nm. In Fig. 2(d), the graph exhibits that detectivity changes according to wavelength. Without a BSF layer, the CCTSe-based photodetector has a detectivity of 5.05 × 10^14^ Jones at wavelength 1150 nm. After adding MoS_2_ as a BSF layer, the detectivity of CCTSe photodetector increases to 2.32 × 10^15^ Jones. The addition of a BSF i.e. MoS_2_ layer improves responsivity by mitigating recombination and promoting better carrier collection, thereby contributing to an overall increase in detectivity [40].

### 3.2 Performance of Cu_2_CdSnSe_4_ photodetector

#### 3.2.1 J-V characteristic curves of CCTSe PD device

**Fig. 3:**
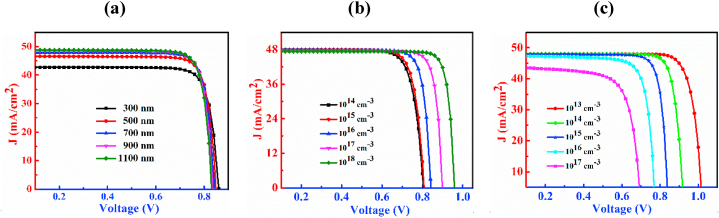
The J-V characteristic plots of n-CdS/p-Cu_2_CdSnSe_4_/p^+^-MoS_2_ PD by varying (a) thickness, (b) doping concentration and (c) defect density.

The J-V characteristic curves of n-CdS/p-Cu_2_CdSnSe_4_/p^+^-MoS_2_ PD by varying thickness, doping concentration, and defect density of CCTSe have been shown in Fig. 3. Fig. 3(a) shows the J-V curves with variation of thickness spanning from 300 nm to 1100 nm of the CCTSe layer. Analyzing the figure, it is noticed that enhancement of the CCTSe layer thickness boosts J_SC_ from 42.68 mA/cm^2^ to 48.84 mA/cm^2^ while slightly declines V_OC_ from 0.86 V to 0.83 V. The relationship between V_OC_ and the dark current in PD is characterized by an inverse relationship. An extension in the absorbing layer thickness tends to enhance recombination of the charge carrier, causing higher dark current and subsequently resulting in a decrease in V_OC_ [41,42]. The J_SC_ generally increases because of the increases in the production of electron and hole pairs due to the growing width of the CCTSe layer [43].

The effect of dopants of the CCTSe semiconductor on the PD is designated in Fig. 3(b). The change in levels of doping span from 10^14^ cm^−3^ to 10^18^ cm^−3^. As a result, the magnitude of J_SC_ is little bit declines from 47.94 mA/cm^2^ to 47.39 mA/cm^2^ but V_OC_ improves significantly from 0.80 V to 0.95 V. This significant increment of V_OC_ because of the production of high built-in potential by the window and absorbing layer interface i.e. n-CdS/p-CCTSe interface [44,45].

The graphical representation of defects of the CCTSe semiconductor in the CCTSe PD is revealed in Fig. 3(c). The bulk flaws of the CCTSe layer is varied from 10^13^ cm^−3^ to 10^17^ cm^−3^. With an increase in defect density, J_SC_ declines from 47.98 mA/cm^2^ to 43.78 mA/cm^2^ and V_OC_ also experiences a significant reduction ranging from 1.02 V to 0.69 V. Because of Shockley-Read-Hall (SRH) recombination, the V_OC_ drops at a significant rate [46]. The J_SC_ drops as more flaws prevent photons from being collected [47]. Generally, when defects are minimal, the carrier diffusion length tends to be high, leading to a low recombination rate and vice versa [48].

#### 3.2.2 QE of CCTSe PD at various parameters of the CCTSe layer

**Fig. 4:**
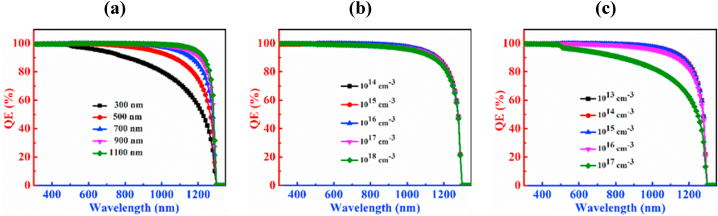
QE characteristic curves of n-CdS/p-Cu_2_CdSnSe_4_/p^+^-MoS_2_ PD by varying (a) thickness (b) doping concentration and (c) defect density.

The QE characteristic curves of the PD with CCTSe as an absorber layer is investigated by changing depth, carrier, and defect levels as depictured in Fig. 4. Rising the thickness of the CCTSe, the absorption capability is increased that is shown in Fig. 4(a). The QE exhibits a peak above 99 % for wavelengths spanning from 300 nm to 480 nm with a 300 nm thick absorber layer. Beyond 480 nm, the QE begins to decline. At a photon wavelength of 1230 nm, the QE drops below 50 %, and at 1300 nm, it reaches zero. For CCTSe absorber layer thicknesses ranging from 700 nm to 1100 nm, slight variations in QE are observed. At a thickness of 1100 nm, the QE remains above 99 % for wavelengths between 300 nm and 500 nm. The photon absorption capability reaches 100 % from 510 nm to 690 nm. However, absorption capability decreases beyond the wavelength of 690 nm reaching zero at 1300 nm. Adjusting absorber the thickness, lifespan and diffusion length of electron-hole pairs in a material can be modified. Increased thickness enhances absorption across both shorter and longer wavelengths, typically attributed to an enhanced extinction coefficient [49].

Fig. 4(b) illustrates that the variation in doping concentration of CCTSe has no discernible effect on the PD device. In this case, the variation of carrier density ranges from 10^14^ cm^−3^ to 10^18^ cm^−3^ and noticed that the QE is above 99 % for wavelength from 300 nm to 500 nm. After 500 nm wavelength, the absorption capability of a photon is 100 % from wavelength 510–590 nm, and beyond wavelength 590 nm the QE decreases. At wavelength 1280 nm, the value of QE is below 50 %, and at 1300 nm it reaches to zero.

Fig. 4(c) describes the upshot of the defects of the CCTSe absorber on the CCTSe PD. It has been seen that there is a noticeable reduction in the photon absorption capability of the CCTSe layer with increasing defect density. Here, the variation of defect density ranges between 10^13^ cm^−3^ to 10^17^ cm^−3^. At a defect density of 10^13^ cm^−3^, the photon absorption capability is above 99 % across the wavelength range of 300–500 nm and the photon absorption capability is 100 % at wavelength of 510–640 nm and it touches zero at a wavelength of 1300 nm. Now, growing defect density to 10^17^ cm^−3^, the QE experiences a rapid reduction. At a defect density of 10^17^ cm^−3^, the photon absorption capability is above 99 % from wavelength ranges between 300 nm and 490 nm and it goes zero at wavelength 1300 nm. The primary cause of this behavior is attributed to the tiny diffusion lengths and increased recombination of charges in the CCTSe material [50].

#### 3.2.3 Responsivity and detectivity of CCTSe PD at various parameters of the absorber layer

**Fig. 5:**
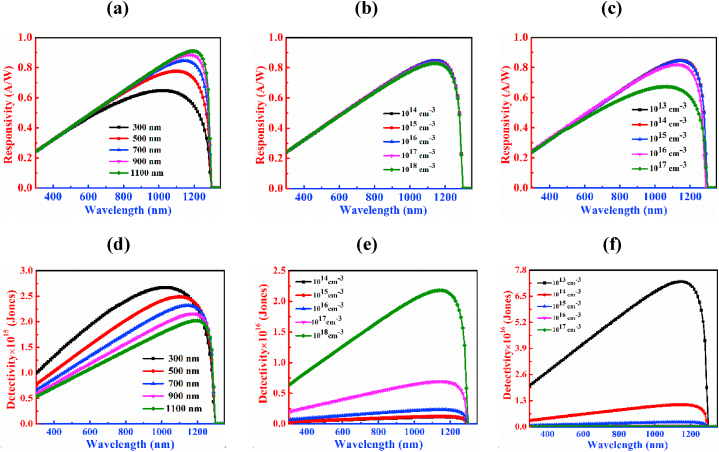
Figures (a)–(c) and (d)–(f) demonstrate the responsivity and detectivity curves of the PD by changing width, carrier, and defects level, respectively of the Cu_2_CdSnSe_4_ absorber.

Fig. 5 provides information about the responsivity and detectivity of the CCTSe PD, showcasing how its performance varies with different parameters such as width, doping concentration, and defect volume of the CCTSe material. The variation of thickness spans from 300 nm to 1100 nm as indicated in Fig. 5(a). By expanding the breadth of the CCTSe semiconductor, the responsivity of the PD escalates from 0.60 A/W to 0.89 A/W at wavelength of 1150 nm. The rise in responsivity with a thicker absorber layer is due to an increased generation of photocurrent resulting from capturing a higher number of photons [51].

Fig. 5(b) displays the effect of the responsivity performance by the variation of acceptor level of the CCTSe layer. It has been displayed that there is an insignificant effect on the PD with the variation of doping concentration in this range i.e. the responsivity remains almost constant at 0.84 A/W at a wavelength of 1150 nm.

Fig. 5(c) depicts the impact of defect density of CCTSe ranging from 10^13^ to 10^17^ cm^−3^ on the CCTSe-based PD. From the figure, it has been displayed that by enhancing the bulk defects of the CCTSe layer, the value of responsivity shrinks from 0.84 A/W to 0.65 A/W. The photodetector's performance is impaired due to heightened recombinations of carrier in the localized energy levels caused by the increased defects [52].

Fig. 5(d) illustrates how changes in the thickness of the absorber layer impact the photodetector's ability to detect the incident light. In the figure, it has been exhibited that detectivity is diminished continuously by the increment of absorber thickness. Here, the variation of absorber width is from 300 nm to 1100 nm, and for this reason, detectivity changes from 2.49 × 10^15^ Jones to 2 × 10^15^ Jones at a wavelength of 1150 nm. This is happened because, detectivity is reversely proportionate to the root of the dark current as shown in equation (5). The decline in detectivity with an enhancing breadth of the absorber in the CCTSe PD is attributed to a higher dark current associated with thicker layer.

Fig. 5(e) indicates significant changes in detectivity with varying doping concentrations of the CCTSe PD at a wavelength of 1150 nm. From the figure, it is sheen that at lower doping concentrations the detectivity is very low, and vice versa. The range of varying doping concentrations of the absorber is from 10^14^ cm^−3^ to 10^18^ cm^−3^ and the changes from 1.14 × 10^15^ Jones to 2.18 × 10^16^ Jones at a wavelength of 1150 nm. This is occurred because, higher doping concentrations reduce dark current, improve signal-to-noise ratio, and consequently increase detectivity in the photodetector [53].

Fig. 5(f) illustrates how detectivity changes with varying defect densities of CCTSe spanning from 10^13^ cm^−3^ to 10^17^ cm^−3^. The changes in detectivity within this range are from 7.23 × 10^16^ Jones to 1.17 × 10^14^ Jones at 1150 nm wavelength. From the above observation, detectivity is higher at low defect densities and vice versa. The occurrence of faster recombination attributed to a higher defects in the CCTSe material, leads to a significant reduction in photocurrent and rise in dark current, consequently, a decrease in detectivity [54].

### 3.3 Performance of Cu_2_CdSnSe_4_ PD with CdS layer

#### 3.3.1 J-V characteristic curves of CCTSe PD by varying physical parameters of CdS layer

**Fig. 6:**
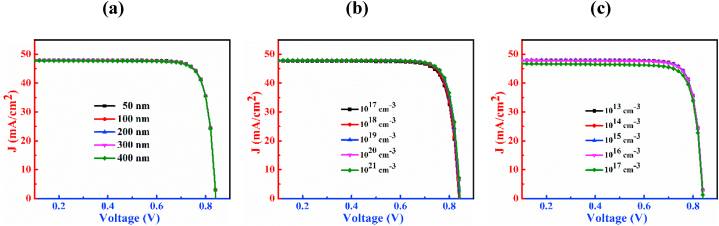
The J-V plots of Cu_2_CdSnSe_4_ PD by varying of (a) width, (b) doping concentration and (c) density of defects of CdS layer.

The impression of the CdS layer on a CCTSe PD by changes in the thickness, doping, and defect density is illustrated in Fig. 6. The thickness of the CdS varies between 50 nm and 400 nm as shown in Fig. 6(a). It depicts that there are no observable changes in J_SC_ and V_OC_ despite manipulating the thickness. The constant values of V_OC_ and J_SC_ are 0.84 V and 47.90 mA/cm^2^, respectively.

Fig. 6(b) displays that by increasing doping concentration in CdS the magnitude of V_OC_ is almost constant at 0.84 V but J_SC_ is collapsing a little bit. Here, the ranges of donor level vary from 10^17^ cm^−3^ to 10^21^ cm^−3^ and the changes of J_SC_ within this range are from 47.94 mA/cm^2^ to 46.84 mA/cm^2^. This is because, increase in the doping concentration can elevate recombination rates, causing electrons and holes to combine and neutralize each other. This phenomenon leads to an overall reduction in photocurrent [53].

Fig. 6(c) exhibits the influence of changes in defect density of the CdS layer on a CCTSe photodetector from 10^13^ cm^−3^ to 10^17^ cm^−3^. The graph depicts a minor variation or fluctuation in J_SC_ but V_OC_ is constant at 0.84 V concerning defect density. When the amount of defects varies from 10^13^ cm^−3^ to 10^17^ cm^−3^, the value of J_SC_ fluctuates from 47.94 mA/cm^2^ to 46.77 mA/cm^2^.

#### 3.3.2 QE characteristics curves of CCTSe PD by varying physical parameters of CdS layer

**Fig. 7:**
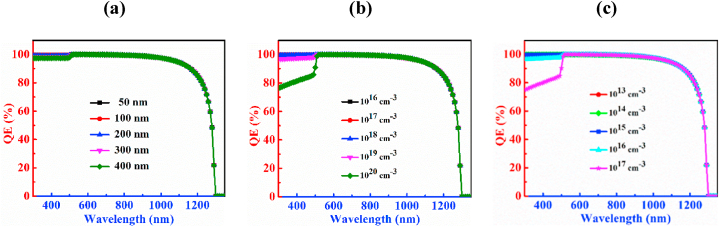
QE characteristic curves of Cu_2_CdSnSe_4_ PD by varying of (a) width, (b) doping concentration and (c) defects of CdS layer.

Fig. 7 displays the mastery of variations in the width, dopant volume, and density of defects of the CdS window layer on the photon absorption capability of a CCTSe photodetector. According to the figure, after the wavelength of 510 nm, there is no discernible effect on PD by varying parameters of CdS. However, at wavelength below 510 nm, there is a significant impact on PD parameters. Fig. 7(a) exhibits the QE plotted against wavelength for varying breadth of the CdS spanning from 0.05 μm to 0.4 μm. The graph illustrates that QE remains consistent and is unaffected by width values exceeding 400 nm. In the range of wavelength 510 nm–590 nm, the material is capable of absorbing 100 % of photons. Interestingly, QE sharply drops to zero at wavelengths longer than 1290 nm. Conversely, at shorter wavelengths, the QE decreases due to buffer or window gain [55].

Fig. 7(b) depicts the range of variation of doping concentration in CdS from 10^16^ cm^−3^ to 10^20^ cm^−3^. In the graph, it has been observed that the impact of doping concentration is constant at a wavelength above 510 nm. At lower doping concentrations, the photon absorption capability of the material is 100 % at wavelengths from 300 nm to 560 nm. After 560 nm, the QE is dropping. On the other hand, at higher doping concentration, the photon absorption capability of the material is 100 % at wavelengths from 520 to 590 nm. QE reaches to zero at wavelengths beyond 1290 nm.

Fig. 7(c) delineates the range of variation in defects of CdS in the range from 10^13^ cm^−3^ to 10^17^ cm^−3^. The graph reveals that the effect of defect density remains constant for wavelengths exceeding 510 nm. At a lower defect density, the material demonstrates 100 % photon absorption capability in the range of 510–590 nm. However, beyond 590 nm, the QE starts decreasing. Conversely, for a higher defect density, the material exhibits 100 % photon absorption capability within the range of 520 nm–590 nm. QE drops to zero at wavelengths beyond 1290 nm. Moreover, it is visualized from the figures that that QE is very high (nearly 100 % or above) in the shorter wavelength range. This may happened owing to the computational error in SCAPS simulation [29].

#### 3.3.3 Responsivity and detectivity of CCTSe PD by varying parameters of the CdS layer

**Fig. 8:**
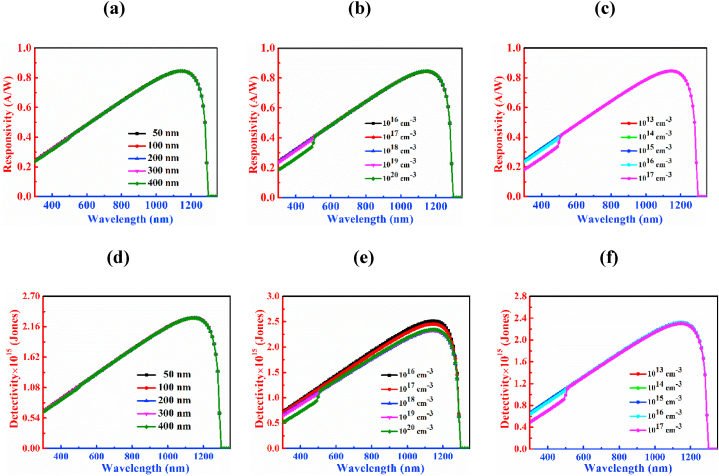
Figures (a)–(c) and (d)–(f) demonstrate the responsivity and detectivity curves by altering width, donor, and defects level, respectively of the CdS.

Fig. 8 demonstrates how the responsivity and detectivity of the CCTSe PD change with variations in CdS parameters. Fig. 8(a) indicates that differences in thickness have a stable dominance on the responsivity operation of the CCTSe photosensor. The highest responsivity value 0.84 A/W is observed at a wavelength of 1150 nm. In Fig. 8(b), varying doping concentrations notably affect the window layer's responsivity, particularly within the 300–500 nm range, showing an inverse correlation between higher doping levels and lower responsivity. Conversely, doping and defects minimally impact the overall responsivity of the heterojunction PD as indicated in Fig. 8(c).

Fig. 8(d) suggests that changes in CdS thickness do not show a significant influence on the detectivity at longer wavelengths of the CCTSe photodetector. Here, the thickness of the CdS layer varies from 50 nm to 400 nm. Noticeably, the detectivity is reported as 2.32 × 10^15^ Jones at a wavelength of 1150 nm. Fig. 8(e) depicts the impacts of donor level on the detectivity curve of the window varies from 10^16^ to 10^20^ cm^−3^. Observing from Fig. 8(e), at higher doping concentrations the value of detectivity is lower and vice versa. At 10^16^ cm^−3^ doping, detectivity is 2.52 × 10^15^ Jones at 1150 nm. Increasing doping to 10^17^ cm^−3^ slightly reduces detectivity to 2.45 × 10^15^ Jones, while at 10^18^ cm^−3^, it further decreases to 2.32 × 10^15^ Jones. Despite this, maintaining a constant detectivity of 2.33 × 10^15^ Jones at 1150 nm, both 10^19^ cm^−3^ and 10^20^ cm^−3^ doping concentrations remain stable in performance. Fig. 8(f) also displays that changes in CdS defect density do not show an effective influence on the detectivity at longer wavelengths of the CCTSe PD. Here, the defect density of the CdS layer varies from ranges between 10^13^ to 10^17^ cm^−3^ and the detectivity is reported as 2.32 × 10^15^ Jones at a wavelength of 1150 nm. The lifetimes of electrons and holes are both 0.1 μs with the dispersion lengths of 5.1 μm and 2.5 μm, respectively in CdS for defect of 10^15^ cm^−3^. The width of the CdS layer is in the range of 0.05–0.4 μm. Consequently, there is nearly no change in performance of PD with CdS thickness. At the same time, as the lifetime and diffusion length of carriers in the CdS layer are quite high compared the thickness, the change in doping and defect have negligible impact on the PD. However, very high doping and defects may have impact on the device operation in practice.

Optimized parameters such as width, doping, and defects volume to 0.1 μm, 10^18^ cm^−3^, and 10^15^ cm^−3^, in turn, achieve a peak responsivity and detectivity of 0.84 A/W and 2.32 × 10^15^ Jones, in turn at photon wavelength of 1150 nm.

### 3.4 Performance of Cu_2_CdSnSe_4_ PD with MoS_2_ layer

#### 3.4.1 J-V characteristics of CCTSe PD by varying physical parameters of MoS_2_ layer

**Fig. 9:**
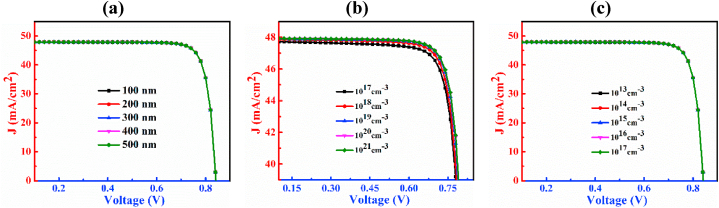
The J-V plots of Cu_2_CdSnSe_4_ PD by altering (a) width, (b) doping level and (c) defects of MoS_2_ layer.

The effects of different MoS_2_ layer widths, doping levels, and flaw densities on a CCTSe PD are shown in Fig. 9. The findings from the figure suggest that these variations do not exert a meaningful impact on the function of the CCTSe PD. Here, the breadth of the MoS_2_ spans from 100 nm to 500 nm (Fig. 9(a)), acceptor density ranges from 10^17^ cm^−3^ to 10^21^ cm^−3^(Fig. 9(b)), and bulk flaws varies from 10^13^ cm^−3^ to 10^17^ cm^−3^(Fig. 9(c)). Despite these wide-ranging variations, the observed values of V_OC_ and J_SC_ remain fixed at 0.84 V and 47.92 mA/cm^2^, respectively.

The lifetime and diffusion path of the charge carrier are quite high compared to the width, doping and defect variation impact of MoS_2_ BSF layer. As a result, nearly no variation in operation of PD is observed owing to the switching of width, acceptor and defect densities of BSF.

#### 3.4.2 QE characteristics of CCTSe PD by varying parameters of MoS_2_ layer

**Fig. 10:**
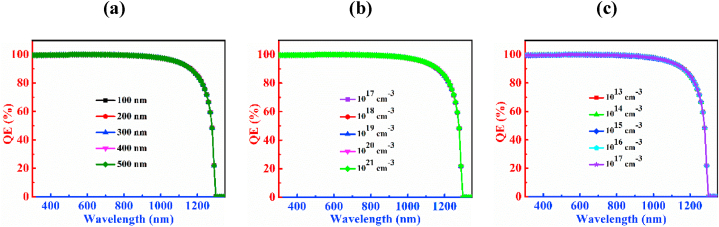
QE characteristic curve of Cu_2_CdSnSe_4_ PD by switching (a) width, (b) doping level and (c) density of defects of MoS_2_ layer.

Fig. 10 indicates the QE characteristic curve of the CCTSe PD, showing the influence of altering the BSF layer's breadth, dopant volume, and defects. The graph expresses those changes in the thickness in Fig. 10(a), doping concentration in Fig. 10(b), and defect density in Fig. 10(c) do not meaningfully affect the functioning of the CCTSe PD. Photon absorption capability is 99.90 % at a wavelength between 300 and 500 nm. After that, the photon absorption capability is 100 % at a wavelength of 510–590 nm. However, below 590 nm, there is an exponential decrease in photon absorption capability and photon absorption capability is 59 % at a wavelength 1270 nm. However, after crossing the 1300 nm, the QE drops to zero photon absorption capability.

However, it is observed that QE is very high (nearly 100 % or above) in the shorter wavelength range in every cases. This may occurred due to the error in computation by SCAPS simulator [29].

#### 3.4.3 Responsivity and detectivity of CCTSe PD by varying parameters of MoS_2_ layer

**Fig. 11:**
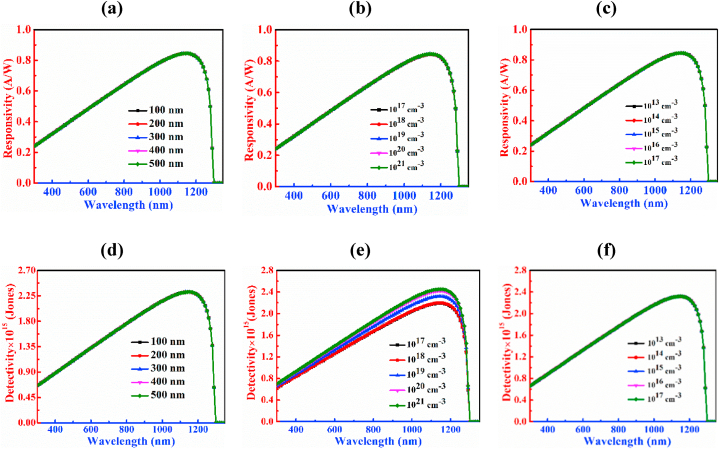
Figures (a)–(c) and (d)–(f) demonstrate the responsivity and detectivity curves, respectively by altering breadth, doping level, and bulk defects of MoS_2_ layer.

In Fig. 11, the responsivity and detectivity of the CCTSe PD are displayed, demonstrating fluctuations in the width, doping, and defect density of MoS_2_ as a BSF. Changes in the thickness revealed in Fig. 11(a), doping delimitated in Fig. 11(b), and defect density shown in Fig. 11(c) of the MoS_2_ layer display negligible impact on responsivity, with a consistent value of 0.84 A/W observed across different parameter values at a wavelength of 1150 nm.

Fig. 11(d) depicts that the range of variation of thickness is between 100 nm and 500 nm. This figure suggests that the variation in thickness within the examined ranges does not noticeably affect the detectivity of the CCTSe PD. Notwithstanding variation in thickness within the test ranges, the detectivity remains constant at 2.32 × 10^15^ Jones at a wavelength of 1150 nm. On the contrary, the doping volume of the BSF material has a role on the detectivity of the CCTSe PD shown in Fig. 11(e).

In the case of doping in the 10^17^-10^18^ cm^−3^, the detectivity of the CCTSe PD is almost close to 2.19 × 10^15^ Jones at wavelength 1150 nm. Raising the acceptor level from 10^18^ 10^21^ cm^−3^ to 10^21^ cm^−3^ results in an escalation in detectivity from 2.19 × 10^15^ Jones to 2.45 × 10^15^ Jones for the CCTSe PD. An escalation in doping concentration, which results in a fall in dark current, contributes to an improvement in detectivity [53]. Fig. 11(f) depicts the range of variation in defect volume from 10^13^ cm^−3^ to 10^17^ cm^−3^. This figure suggests that the variation in defects amid the examined range does not noticeably influence the detectivity of the CCTSe PD and the detectivity remains constant at 2.32 × 10^15^ Jones at a wavelength of 1150 nm.

### 3.5 Resistances and temperature effect on CCTSe PD

**Fig. 12:**
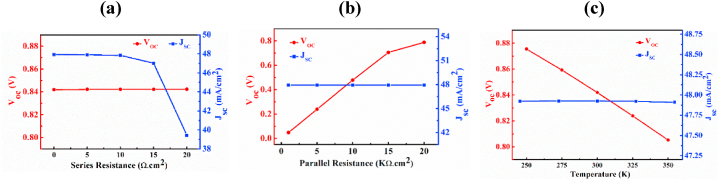
The (a) series, (b) parallel resistance and (c) temperature effects on V_OC_ and J_SC_ of n-CdS/p-Cu_2_CdSnSe_4_/p^+^-MoS_2_ Photodetector.

Fig. 12 illustrates the masteries of series and parallel resistance, as well as temperature, on the CCTSe PD. The magnitude of series and shunt resistance is zero and infinite, respectively in an ideal condition [50]. However, in practice, series resistances often result from contact resistance between different layers, like the front and back contact metal. Shunt resistances are typically caused by recombination in defect states, and interestingly they tend to increase as defect density decreases [50].

Fig. 12(a) indicates that there has no role of varying the resistance in series on V_OC_ as it stays at 0.82 V, but in the case of J_SC_, it decreases a little bit from 47.92 mA/cm^2^ to 47.01 mA/cm^2^ when series resistance rises from 0 to 15 Ω cm^2^. After 15 Ω cm^2^, there is a noticeable decrease in J_SC_ and the value of J_SC_ is recorded as 39.44 mA/cm^2^ at series resistance of 20 Ω cm^2^. Fig. 12(b) shows that there has no impact of varying parallel resistance on J_SC_ i.e. the J_SC_ is fixed at 47.92 mA/cm^2^ but in case of V_OC_, it has noticeable effect of varying parallel resistance. By increasing parallel resistance, the value of V_OC_ is also increasing. The parallel resistance varies within the range of 1–20 kΩ cm^2^, resulting in a variation of the V_OC_ from 0.05 V to 0.79 V. The observations suggest that higher shunt and lower series resistance contribute to enhanced PD performances [50].

Fig. 12(c) delimitates the impact of device temperature of the PD performance. It has been exhibited that the J_SC_ of the PD remains constant at 47.92 mA/cm^2^. But in the case of V_OC_, it decreases with rise in temperature. Due to the temperature rises, the reverse saturation or dark current (J_0_) tends to growth exponentially. This escalation in the dark current contributes to a higher dark current in the heterojunction cell, executing a reduction in the V_OC_ from almost 0.87 V–0.80 V, according to the Shockley diode equation [42,44]:

(7) $$J=J_{L}-{\;J}_{0}\left(\exp\left(\frac{qV}{kT}\right)-1\right)$$

where, J is the total current through the structure, J_L_ is the light-produced current, J_0_ refers to the reverse saturation current, q indicates charge of elements, V denote the voltage, k stands for the Boltzmann constant and T indicates the temperature measured on the absolute scale.

This temperature-dependent behavior suggests that while the photogenerated current remains relatively stable, the voltage output decreases at higher temperatures. Therefore, a temperature of 300 K is intentionally selected to minimize this decline.

### 3.6 C-V analysis of the CCTSe PD

The built-in potentials (***ψ***_bi_) at the n-CdS/p-CCTSe and p-CCTSe/p^+^-MoS_2_ heterointerfaces have been estimated by analyzing the capacitive (C-V) response of the Mott-Schottky relation [56]. The point where the 1/C^2^-V graph intersects the y-axis are utilized to estimate the built-in voltage using the following equation [56]:

(8) $$\frac{1}{C^{2}}=\frac{c\left(\frac{kT}{q}\;-\;\psi_{bi}\;-\;V\right)}{qA^{2}\in_{0}\in_{CCTSe}N_{D}}$$

wherein, the symbol $\in_{0}\in_{CCTSe}$ is the permittivity of CCTSe, V represents voltage, and A represents the diode area.

**Fig. 13:**
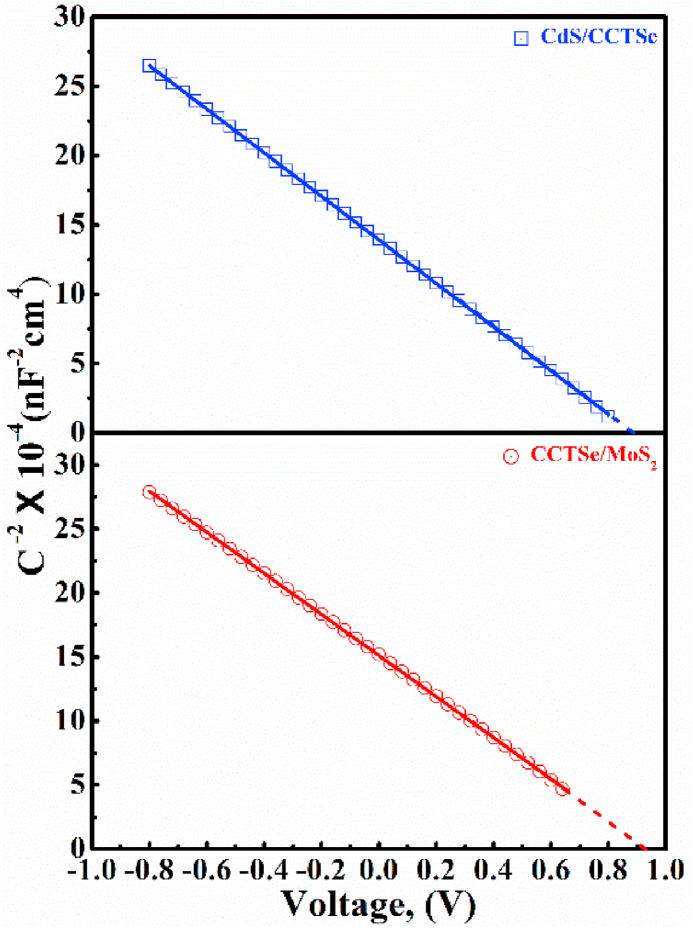
C-V analysis of the proposed n-CdS/p-CCTSe/p^+^-MoS_2_ PD gadget.

Fig. 13 illustrates the 1/C^2^−V plots for the n-CdS/p-CCTSe and p-CCTSe/p^+^-MoS_2_ junctions. The ***ψ***_bi_ is derived by determining the intercept opint of the voltage-axis by matching and projecting the straight regions of the lines in equation (8). The predicted ***ψ***_bi_ values for the n-CdS/p-CCTSe and p-CCTSe/p^+^-MoS_2_ interfaces are 0.87 V and 0.92 V, respectively. The total potential of the device can be expressed by summing the above two vlues. The precise band alignment between the n-CdS/p-CCTSe and p-CCTSe/p^+^-MoS_2_ is responsible for obtaining this higher ***ψ***_bi_ of 1.79 eV.

### 3.7 Overall comparison

The comparison between with MoS_2_ and without the MoS_2_ layer revealed in Table 2 specifies that without the BSF layer i.e. the values of J_SC_, V_OC_, responsivity, and detectivity at 1150 nm wavelength of light are 45.57 mA/cm^2^, 0.76 V, 0.72 A/W and 5.05 × 10^14^ Jones, respectively. Now, by adding the MoS_2_ layer the values of J_SC_, V_OC,_ responsivity, and detectivity at 1150 nm wavelength of light are 47.92 mA/cm^2^, 0.84 V, 0.84 A/W, and 2.32 × 10^15^ Jones, respectively. So, the insertion of the BSF layer has demonstrated an increase in all parameters.

**Table 2:** The responsivity and detectivity of CCTSe PD with and without BSF layer.

Device Structure	Photocurrent (mA/cm^2^)	Voltage (V)	Responsivity (A/W)	Detectivity (Jones)	Wavelength (nm)
n-CdS/p-Cu_2_CdSnSe_4_	45.57	0.76	0.72	5.05 × 10^14^	1150
n-CdS/p-Cu_2_CdSnSe_4_/p^+^-MoS_2_	47.92	0.84	0.84	2.32 × 10^15^	1150

Table 3 presents a comparative analysis between the proposed CCTSe-based photodetector and other thin film photodetectors. The results indicate that the novel CCTSe structure exhibits superior responsivity and detectivity compared to the alternative PDs listed in the table. These findings suggest that the CCTSe-based photodetector holds potential for advancing research in photodetection.

**Table 3:** Comparison among different photodetectors.

Material with structure	Types	Responsivity (A/W)	Detectivity (Jones)	References
CZTSSe based	Simulation	0.018	1 × 10^9^	[12]
CZTS based	Simulation	0.41	1 × 10^12^	[13]
CZTSSe/CdS-based hetero-junctions	Simulation	0.39	2.04 × 10^11^	[14]
CH_3_NH_3_PbI_3_/GaN heterojunction based	Experimental	0.198	7.96 × 10^12^	[57]
SnSx/TiO_2_ heterojunction based	Experimental	0.0065	7.26 × 10^10^	[58]
CCTSe based	simulation	0.84	2.32 × 10^15^	This work

## 4 Conclusion

The article discusses the modeling and computation of a CCTSe-based n-CdS/p-Cu_2_CdSnSe_4_/p^+^-MoS_2_ PD. The work explores the role of various parameters, including width, carrier, and defects level in the window, BSF, and absorber layers in CCTSe PD. The window, BSF, and absorber layer optimum thickness parameters are 100 nm, 200 nm, and 700 nm, respectively. The optimized doping concentration values for the window, BSF, and absorber layers are 10^18^, 10^19^, and 10^16^ cm^−3^, respectively. Likewise, the defect density values for these layers have been optimized at 10^15^ cm^−3^ for all cases. This heterojunction photodetector shows a very higher J_SC_ of 47.92 mA/cm^2^ and V_OC_ of 0.84 V and promising photo response under AM 1.5G illumination. By investigating all parameters, the highest value of responsivity and detectivity recorded as 0.84 A/W and 2.32 × 10^15^ Jones, respectively at a photon wavelength of 1150 nm. This material proves to be a suitable fit for optoelectronic applications, particularly excelling in achieving higher responsivity and detectivity values.

## Notes

The authors have none known competing ﬁnancial interest.

## Data availability

Data of the work will be available from the corresponding author upon resonable request.

## CRediT authorship contribution statement

**Md. Choyon Islam:** Writing – original draft, Validation, Investigation, Formal analysis, Data curation. **Bipanko Kumar Mondal:** Writing – original draft, Visualization, Validation, Methodology, Formal analysis. **Md. Alamin Hossain Pappu:** Writing – original draft, Visualization, Validation, Methodology, Investigation, Formal analysis. **Jaker Hossain:** Writing – review & editing, Writing – original draft, Visualization, Validation, Supervision, Methodology, Formal analysis, Conceptualization.

## Declaration of generative AI and AI-assisted technologies

The authors did not utilize any AI or AI-assisted technology for the preparation of this manuscript.

## Declaration of competing interest

The authors declare that they have no known competing financial interests or personal relationships that could have appeared to influence the work reported in this paper.
